# Supplementary material for: Regulative Loop between β-catenin and Protein Tyrosine Receptor Type γ in Chronic Myeloid Leukemia
Source: Int J Mol Sci. 2020 Mar 26;21(7):2298. doi: 10.3390/ijms21072298 (PMC7177637; doi:10.3390/ijms21072298)
Supplement: Supplementary file 1 [file ijms-21-02298-s001.pdf]

SUPPLEMENTARY TABLES

Target	size (bp)	Forward primer (5'-3')	Reverse primer (5'-3')
**PTPRG**	288	GTCACCAGTCTCCTATTGAC	AAATCTGCATCTCAACAGGA
**β-Catenin**	235	CAACTAAACAGGAAGGGATGG	ATGACGAAGAGCACAGATGG
**MYC**	203	CTTCTCTCCGTCCTCGGATTCT	GAAGGTGATCCAGACTCTGACCTT
**p21/WAF1**	272	CCAGCATGACAGATTTCTACCA	GAACCTCTCATTCAACCGCC
**Cyclin D1**	177	GCGGAGGAGAACAAACAGAT	TGAGGCGGTAGTAGGACAGG
**DNMT1**	201	TCTTTGATGCCAACGAGTCTG	CCATTAACACCACCTTCAAGAG
**PTPRG prom for chIP PCR**	606	TGAAAGCCCGAAAAAGAAAA	TGGATCCTGGCAGAGACTTT
**PTPRG prom for Sequencing**	898	GGTAGTTGTTAGGAAATTAGTT	ATACCTCCATACAAAAAATAACAT
**GATA1**	175	ATTCAGCAGCCTATTCCTCTCC	CTGTTCTGCCCATTCATCTTGT
**β-Actin**	290	TGACCCAGATCATGTTTGAG	CTTCTCCTTAATGTCACGCAC

Supplementary table 1 – qRT–PCR primers designed with the free open-source GUI application PerlPrimer v1.1.21

Antibody	**Host**	**Company**
tot-β-Catenin B-9	Mouse	Santa Cruz
tot-β-Catenin [E247]	Rabbit	Abcam
phospho-β-Catenin (Tyr 654)	Rabbit	ECM Biosciences
phospho-BCR-ABL1 (Tyr 245)	Rabbit	Cell Signaling
tot-BCR-ABL1	Rabbit	Cell Signaling
DNMT1	Rabbit	Proteintech
DNMT3a	Rabbit	Genetex
DNMT3b	Rabbit	Genetex
Cyclin D1	Rabbit	Genetex
p21/WAF1	Mouse	Genetex
Tubulin	Mouse	MilliporeSigma
Lamin A/C	Rabbit	Genetex
Axin1	Rabbit	Genetex
β-Actin	Rabbit	MilliporeSigma

Supplementary table2 –Western Blotting antibodies
